# Recurrent Falls in People with Parkinson's Disease without Cognitive Impairment: Focusing on Modifiable Risk Factors

**DOI:** 10.1155/2014/432924

**Published:** 2014-11-23

**Authors:** Lorena R. S. Almeida, Guilherme T. Valença, Nádja N. Negreiros, Elen B. Pinto, Jamary Oliveira-Filho

**Affiliations:** ^1^Movement Disorders Clinic, Bahia State Health Attention Center for the Elderly, Avenida ACM, s/n, Ed. José Maria de Magalhães Netto, Iguatemi, 41820-000 Salvador, BA, Brazil; ^2^Post-Graduate Program in Health Sciences, Federal University of Bahia, Largo do Terreiro de Jesus, s/n, Centro Histórico, 40025-010 Salvador, BA, Brazil

## Abstract

Falls can be considered a disabling feature in Parkinson's disease. We aimed to identify risk factors for falling, testing simultaneously the ability of disease-specific and balance-related measures. We evaluated 171 patients, collecting demographic and clinical data, including standardized assessments with the Unified Parkinson's Disease Rating Scale (UPDRS), activities of daily living (ADL) and motor sections, modified Hoehn and Yahr Scale, Schwab and England, eight-item Parkinson's Disease Questionnaire, Activities-specific Balance Confidence Scale, Falls Efficacy Scale-International (FES-I), Berg Balance Scale, Dynamic Gait Index, Functional Reach, and Timed Up and Go. ROC curves were constructed to determine the cutoff scores for all measures. Variables with *P* < 0.1 entered a logistic regression model. The prevalence of recurrent falls was 30% (95% CI 24%–38%). In multivariate analysis, independent risk factors for recurrent falls were (*P* < 0.05) levodopa equivalent dose (OR = 1.283 per 100 mg increase; 95% CI = 1.092–1.507), UPDRS-ADL > 16 points (OR = 10.0; 95% CI = 3.6–28.3), FES-I > 30 points (OR = 6.0; 95% CI = 1.6–22.6), and Berg ≤ 48 points (OR = 3.9; 95% CI = 1.2–12.7).We encourage the utilization of these modifiable risk factors in the screening of fall risk.

## 1. Introduction

Parkinson's disease (PD) is a common neurodegenerative disorder in older adults and postural instability is well known to occur in these individuals, increasing the risk for falling [1–4]. Falls are one of the most disabling features of PD and the incidence of falls is high, with reported rates varying from 35 to 68% for one or more falls [5–12] and from 24 to 43% for two or more falls [5, 7, 10, 12, 13], in a 3- to 12-month follow-up period.

The consequences of falls are numerous and can affect not only fallers, but also their family and community. Falls can result in injuries such as head trauma and hip fractures and may also contribute to increased fear of falling, reduced level of activity, and functional impairment, leading to decreased quality of life in these individuals [14–16]. The financial cost of a fall is high, for patients and the health system, as the fall-related injuries, especially the fractures, are a common reason of hospitalization [17].

Several risk factors of falls have been proposed, although we can note that some of them have limited use in the general clinical practice, such as longer disease duration and dementia [8]; abnormal posture, freezing of gait (FOG), and frontal impairment [9]; and female gender, older age, and symmetrical onset [18]. Previous authors have found poor balance confidence and longer Timed Up and Go Test (TUG) as factors related with falls [19], reflecting the role of balance-related measures in fall risk assessment. Some functional activities have been described as common situations of falls in people with PD, such as transfers, bending or reaching for an object, turning, and walking [14], and these tasks are addressed by some balance scales [20–25].

In a meta-analysis of prospective studies of falling in PD, the best predictor of falls was the presence of two or more falls in the previous year [26]. Although this is important for predicting future falls, this is not a modifiable risk factor and does not help to identify patients at risk before their first fall.

Considering the wide range and scope of falls consequences, it is of paramount importance to increase our knowledge of factors associated with recurrent falls, in particular, those identifiable through common clinical measures. This will provide useful information for identification of individuals at risk of falling and may contribute to the development of specific interventions to prevent falls.

The goals of this study were to report the frequency of falls in a sample of community-dwelling individuals with PD and to identify risk factors for recurrent falls, testing simultaneously the ability of disease-specific and balance-related measures to predict falls.

## 2. Methods

### 2.1. Participants

The study sample consisted of consecutive individuals with PD with independent walking ability who attended the Movement Disorders Clinic at the State of Bahia Centre for the Elderly Health Attention, Brazil. Independent walking ability was defined when the patient was able to walk without assistance of another person, with or without a walking aid. The movement disorders clinic is a reference service for the state of Bahia, where approximately 500 elderly (≥60 years old) patients with PD and other parkinsonian syndromes are followed, mostly from the public health system. The diagnosis of idiopathic PD was made by a certified neurologist, in accordance with the UK Parkinson's Disease Society Brain Bank Diagnostic Criteria [27]. Exclusion criteria were other neurological disorders than PD, cognitive impairment (assessed by the mini-mental state examination and based on determined cutoff scores by previous authors, in accordance with the level of education of each participant) [28], severe visual disturbance, vestibular dysfunction, and musculoskeletal problems that might affect balance.

Patients were recruited between April of 2010 and January of 2012. The study was approved by the local research ethics committee. Initially, potential participants were approached by a physical therapist (L.R.S.A) and explanations related to the study were given to them. After that, each individual who decided to participate in the study provided written informed consent.

### 2.2. Assessments

Demographic and clinical data were recorded including the following information: age, gender, disease duration, history of falls, antiparkinsonian medications taken, presence of motor fluctuations, dyskinesia, FOG, and comorbidities (cardiovascular diseases, such as high blood pressure, heart failure, arrhythmia, hypercholesterolemia, peripheral vascular diseases, and diabetes; depression; urinary incontinence; constipation; osteoporosis). The definition of each comorbidity was based on self-report complemented by chart review. The levodopa equivalent dose (LED) was calculated based on a previously reported conversion factor [29]. A fall was defined as “an event which results in a person coming to rest unintentionally on the ground or other level, not as the result of a major intrinsic event or overwhelming hazard” [30]. Subjects were classified as nonfallers if they reported none or one fall in the preceding 12 months and as recurrent fallers if they reported two or more falls in the preceding 12 months. This group division was performed due to evidence supporting differences on clinical features and fall characteristics between individuals with PD who had experienced a single fall and those who are recurrent fallers and similarities between individuals with PD with no falling history and only a single fall [11].

### 2.3. PD-Specific Scales

The UPDRS was completed, including the activities of daily living (ADL) and motor sections, to assess both motor disability and motor impairment. The ADL section ranges from 0 to 52 points (greater disability) and the motor section from 0 to 108 (greater impairment) [31]. The modified Hoehn and Yahr scale (H&Y) was used to evaluate the stage of PD (stage 5 means wheelchair-bound or bedridden) [32], and the Schwab and England scale (S&E) to measure capacity for daily living (100% indicates a completely independent person) [33]. The eight-item Parkinson's Disease Questionnaire (PDQ-8) was used to measure quality of life and is scored 0 to 100 (worst) [34]. The UPDRS was administered by a neurologist (G.T.V.) or physical therapist (L.R.S.A.), previously trained for this assessment.

### 2.4. Balance-Related Measures

Fear of falling was evaluated with two scales: the Activities-specific Balance Confidence Scale (ABC), which measures self-perceived balance confidence for completing daily living tasks, ranging from 0 to 100% (full confidence) [15, 20], and the Falls Efficacy Scale-International (FES-I), which assess concern about falling while performing certain activities of daily living, ranging from 16 to 64 points (highest concern) [21].

Balance and gait were assessed with four tests: Berg Balance Scale (BBS) [22], Functional Reach Test (FRT) [23], Timed Up and Go Test (TUG) [24], and Dynamic Gait Index (DGI) [25]. The BBS is an assessment tool for static and dynamic balance, which consists of 14 tasks related to common movements of daily living, such as transfers, turning, and balancing on reduced base of support, and is scored 0 to 56 points (best) [22, 35]. The FRT is used to measure anteroposterior stability while standing with a stable base of support, recorded in centimeters [23, 36]. The TUG assesses balance and functional mobility, in which patients arise from an armchair, walk for 3 meters, turn, walk back to the chair, and sit down, with results recorded in seconds. Patients were allowed to wear their regular footwear and use their customary walking aid [24, 37]. The DGI quantifies dynamic balance during gait, including tasks such as walking forward, with head turns and changing gait speed, ranging from 0 to 24 points (best) [25, 38].

All patients were assessed in the movement disorder clinic on the same occasion as they were recruited. They were evaluated during the “on” phase of the medication cycle and the balance measures were administered in the order described above by a trained physical therapist (L.R.S.A.). Participants were allowed to rest as needed during the balance assessments. Only the TUG was performed twice, and the first trial was considered as practice [24]. All the measurements took approximately 60 minutes to be performed.

### 2.5. Statistical Analysis

Statistics were performed using SPSS for Windows (version 16.0). Descriptive statistics were calculated for demographic and clinical variables, while comparison of recurrent fallers and nonfallers was completed using the Mann-Whitney test for continuous variables and the Pearson's chi-square test for categorical variables. To identify collinearity between the functional capacity scales, the fear of falling measures, and the balance tests, the Spearman correlation was performed and a value of *r* ≥ 0.75 was set. Rates of falling were reported with 95% confidence intervals (CI). A significance level of 0.05 was set for these statistical tests.

Variables with *P* < 0.1 were selected to enter into the multivariate regression analysis. To improve the clinical utility of the disease-specific, fear of falling, and balance measures, receiver operating characteristic (ROC) curves were constructed to determine the cutoff scores that would be best related to recurrent fallers, based on Youden indices [39]. The area under the curve (AUC), sensitivity, and specificity were determined for each test, as well as the 95% CI. These measures were entered into the multivariate regression model with the cutoff scores previously chosen, as dichotomous variables. A backward stepwise (likelihood ratio-based) logistic regression was carried out, with falls history as the dependent variable (0 = no falls or one fall; 1 = two or more falls). A significance level of 0.05 was set for variables remaining in the model. The proportion of variance explained by the logistic regression model was estimated by Nagelkerke's pseudo-*R*-squared.

## 3. Results

During the recruitment period, a total of 515 people attended the movement disorders clinic and 171 individuals with PD were enrolled in the study. One hundred and ninety-one patients were excluded because they did not meet the inclusion criteria for the following reasons: atypical parkinsonism (*n* = 58), essential tremor (*n* = 73), dystonia (*n* = 56), spinocerebellar ataxia (*n* = 2), and chorea (*n* = 2). One hundred and two people with PD were excluded due to other neurological conditions in addition to idiopathic PD (*n* = 10), cognitive impairment (*n* = 32), musculoskeletal problems that affected balance (*n* = 15), severe visual disturbance (*n* = 3), and vestibular dysfunction (*n* = 5) and because they were unable to walk independently (*n* = 37). An additional 51 individuals with PD were approached, but 16 were not regularly taking antiparkinsonian medication and 35 were unwilling to consent to participate in the study. This number (*n* = 51) corresponds to 23% of all participants who met all the eligibility criteria (*n* = 222).

The final sample consisted of 171 patients. The median age was 70 years (range 60–94) and 89 (52%) were female. Patients had a median H&Y stage of 2.5 (range 1.0–4.0) and the median LED was 600 mg (range 300–1621.9). Seventy-four (43%, 95% CI 36%–51%) patients reported one or more falls in the previous 12 months. Most patients (30%, 95% CI 24%–38%) reported two or more falls and were classified as recurrent fallers.

### 3.1. Comparison between Recurrent Fallers and Nonfallers

Demographic and disease-specific characteristics of the sample are shown in Table 1. Recurrent fallers had longer disease duration and greater disease severity according to H&Y stage, UPDRS motor score, and LED. They had functional limitations based on the UPDRS ADL and S&E scale, and lower scores on the PDQ-8. Recurrent fallers also had worse performance on the BBS, DGI, FRT, and TUG, as well as higher degree of fear of falling based on FES-I and ABC (*P* < 0.001), as shown in Table 1. Dyskinesia (*P* = 0.001), FOG (*P* < 0.001), urinary incontinence (*P* < 0.001), and constipation (*P* = 0.002) were associated with recurrent falls. Motor fluctuations were not associated with recurrent falls, but we found a borderline *P* value of 0.084.

### 3.2. ROC Analysis

The chosen cutoff scores and validity indices were as follows for the disease-specific measures: H&Y > 2.5 (AUC = 0.77, 95% CI 0.70–0.83; sensitivity = 0.67, 95% CI 0.53–0.80; specificity = 0.76, 95% CI 0.67–0.83); UPDRS-motor > 33 points (AUC = 0.78, 95% CI 0.71–0.84; sensitivity = 0.80, 95% CI 0.66–0.90; specificity = 0.72, 95% CI 0.63–0.80); S&E ≤ 70% (AUC = 0.77, 95% CI 0.70–0.83; sensitivity = 0.58, 95% CI 0.43–0.71; specificity = 0.86, 95% CI 0.79–0.92); and UPDRS-ADL > 16 points (AUC = 0.86, 95% CI 0.80–0.91; sensitivity = 0.67, 95% CI 0.53–0.80; specificity = 0.92, 95% CI 0.85–0.96).

In relation to the fear of falling scales, the cutoff scores and the corresponding values were FES-I > 30 points (AUC = 0.81, 95% CI 0.74–0.87; sensitivity = 0.88, 95% CI 0.76–0.96; specificity = 0.66, 95% CI 0.57–0.75) and ABC ≤ 46% (AUC = 0.83, 95% CI 0.76–0.88; sensitivity = 0.71, 95% CI 0.57–0.83; specificity = 0.79, 95% CI 0.71–0.86).

Regarding the balance measures, the determined cutoff scores and validity indices were BBS ≤ 48 points (AUC = 0.86, 95% CI 0.80–0.91; sensitivity = 0.81, 95% CI 0.67–0.90; specificity = 0.73, 95% CI 0.64–0.81); DGI ≤ 18 points (AUC = 0.84, 95% CI 0.77–0.89; sensitivity = 0.79, 95% CI 0.65–0.89; specificity = 0.74, 95% CI 0.65–0.82); FRT ≤ 17 centimeters (AUC = 0.79, 95% CI 0.72–0.85; sensitivity = 0.75, 95% CI 0.61–0.86; specificity = 0.76, 95% CI 0.68–0.84); TUG > 16.6 seconds (AUC = 0.76, 95% CI 0.69–0.82; sensitivity = 0.69, 95% CI 0.55–0.81; specificity = 0.76, 95% CI 0.68–0.84).

### 3.3. Multivariate Logistic Regression Model

In the multivariate analysis, 164 patients were included due to missing data related to the UPDRS motor score, which was entered into the model. The H&Y score and the LED were also used as measures of disease severity rather than disease duration. Collinearity was found between the UPDRS-ADL and S&E scale, and the first one was selected to enter into the model due to its higher AUC. To remove redundancy, FOG, also used as a dichotomous variable, was excluded from the model as it is an item from the UPDRS-ADL. As both the ABC and FES-I scales assess fear of falling during everyday tasks and they had similar values of AUC, we chose to enter the FES-I into the model, due to its higher value of sensitivity. As we found collinearity between all balance measures, the BBS was entered into the model due to its higher AUC.

The independent risk factors for recurrent falls in the multivariate model were LED, UPDRS-ADL > 16 points, FES-I > 30 points, and BBS ≤ 48 points (Table 2). The final model explained 64.6% of the variance of the history of recurrent falls.

## 4. Discussion

This study aimed to report the frequency of falls in a sample of individuals with PD and to identify risk factors for recurrent falls. The reported frequency of one or more falls was 43% and of two or more falls 30%. These findings confirm that falling is a common feature of PD. When analyzing retrospective studies, our reported frequency of one or more falls was lower than that found by Landers et al. (51%) [40] and Dibble and Lange (55%) [41], but the frequency of recurrent falls was similar to that obtained by Leddy et al. (31%) [42]. As expected, our rate of falling was lower than that found by the majority of prospective studies (45–68%) [5–10, 12]. However, it is of interest to note that, regarding recurrent falls, our rate was similar to that obtained by Duncan et al. (32%) [13], lower than those reported by Gray and Hildebrand (41%) [5] and Cole et al. (43%) [12], but higher than that related by Kerr et al. (24%) [10] and Bloem et al. (25%) [7]. These differences may be due to different follow-up periods and characteristics of the sample in terms of disease severity.

Our main results were the independent association between recurrent fallers and higher LED and the association between recurrent fallers and three clinical scales (BBS, FES-I, and UPDRS-ADL). Recurrent fallers had longer disease duration and increased disease severity based on the H&Y and UPDRS motor sections than nonfallers, as well as higher LED. Recurrent fallers also showed reduced quality of life, higher degree of fear of falling and functional impairment, and decreased performance on all balance measures. These results are in agreement with those reported in previous studies [10, 11, 40, 41]. Together, these results suggest that the LED (corresponding with disease severity), BBS ≤ 48 points (showing postural instability), FES-I > 30 points (representing fear of falling), and UPDRS-ADL > 16 points (indicating functional impairment) could help identify patients at a higher risk of falling as those with this profile seem to be at risk for becoming or being recurrent fallers.

Previous research found that H&Y stage, UPDRS motor score, and disease duration were considered risk factors for falls in multivariate analyses [7, 8, 10, 43, 44]. In this study, the LED was a factor related to falls. Kerr et al. [10] found that levodopa medications were slightly correlated with postural sway on the firm surface when all patients were considered, but there were no correlations when analyzing fallers and nonfallers separately. Previous studies have reported that postural instability is not sufficiently responsive to dopamine replacement due to the involvement of nondopaminergic pathways in gait and balance dysfunction [4, 45, 46], which can explain the higher level of balance impairment in individuals with PD who are recurrent fallers, even with higher levodopa dosages. The LED remained an independent risk factor for falling, probably because of its relation with longer PD duration, which is typically characterized by greater disease severity and postural instability, characteristics significantly higher in recurrent fallers.

The UPDRS-ADL > 16 points was also found to be an independent risk factor for recurrent falls. This subscale comprises some activities that can be difficult to perform not only because of motor limitations, but also due to postural instability, such as dressing and hygiene. Moreover, it rates walking difficulty and occurrence of FOG, factors linked to balance impairment, in addition to frequency of falling. FOG is a disabling feature of PD that occurs more often with disease progression and is related to falls in these patients [5, 47]. The presence of FOG as well as scores on the FOG questionnaire has been considered independent related to falls in multivariate models [9, 10, 43]. Matinolli et al. [48] in a two-year prospective study about falls and mortality in PD also described the UPDRS-ADL as an independent risk factor for falling. Kerr et al. [10] in a six-month prospective study about predictors of falls in PD suggested a multivariate model including the UPDRS total score but reported that it can be substituted by the UPDRS-ADL, as they obtained similar sensitivity and specificity when this score was used. There are reliable formulas for transforming scores from the UPDRS-ADL to the recent developed MDS-UPDRS scale [49].

The BBS, with a cutoff score of ≤ 48 points, remained an independent risk factor for falling. The BBS is a well-established tool to assess postural instability in people with PD [50–52] and previous studies have shown its greatest overall ability for predicting falls when compared with the other scales used in this study [40, 41]. Previous authors used different tools to evaluate postural instability, such as the Romberg test, Tinetti index, retropulsion test, and measurements of postural sway [7–10, 53], which can explain the diversity of results about the clinical validity of balance tests to predict falls in individuals with PD. The Tinetti total score remained a predictor of falls in the multivariate model suggested by Kerr et al. [10] and its balance subscale was independently associated with falls in Contreras and Grandas' Study [44]. Lim et al. [43] and Mak and Pang [19] proposed a model including the TUG as an independent risk factor for falls, but it was the only balance test included in their study.

Recurrent fallers had higher degree of fear of falling on both ABC and FES-I. ABC scores have been determined to be independently associated with increased risk of falling in individuals with PD [19, 54]. In accordance with these findings, we showed that a FES-I > 30 points, reflecting fear of falling, was an independent risk factor for falling. It has been shown that fear of falling correlates with performance-based balance and mobility tests, as well as with functional capacity and disease severity [16], factors related to falls. It is important to note that this fear of falling may be protective if it increases caution during performance in daily activities, but it can be a negative factor when it leads to restrictions of mobility and social isolation, contributing to functional decline and to an actual increase in the risk of falling [16, 19].

Other variables were associated with recurrent falls, although they were not found to be independent risk factors in the presence of the other factors included in our analysis. Recurrent fallers presented more dyskinesia than nonfallers. This high frequency of dyskinesia could be explained by the higher intake of levodopa showed by recurrent fallers [1]. It has been shown that dyskinesia may contribute to postural instability in advanced PD [55] and previous authors also found that dyskinesia was associated with falls [10, 53]. We believe that the presence of dyskinesia could contribute to an increased postural sway, especially when the trunk and feet are affected.

Urinary incontinence and constipation, both considered signs of autonomic dysfunction in PD, were associated with falls. Although we have not investigated thoroughly urinary incontinence, it is known that nocturia and urinary urgency are common events in PD [1]. Usually during the night, individuals with PD are without the effect of levodopa, which may generate greater motor difficulties and risk of falling when standing and walking to the toilet. Balash et al. [56] in a study about falls with 350 patients with PD identified urinary incontinence as an independent risk factor for being a recurrent faller. The association between constipation and falls is discussed in clinical practice probably due to its contribution to decreased absorption of levodopa [57].

Motor fluctuations might be associated with postural instability and higher risk of falling, as it generates reduction of motor performance [58], leading to functional impairment and gait difficulty. Some authors have found association between falls and motor fluctuations [8, 44], whereas others have reported only borderline *P* value [53], similar to our findings.

The main strength of our study was to simultaneously test disease-specific and balance-related measures in the same sample population, suggesting the importance of using common clinical scales to assess risk of falling. The utilization of the scales that we found to be associated with recurrent falls, with their respective cutoff scores, may be useful in clinical practice. These scales provide detailed information about impaired functional activities and balance-related tasks and can be used not only in the assessment of the individuals with PD, but also during treatment, and to evaluate treatment outcomes. Our results also provide evidence for an independent validation sample using these scales, selecting the best combination of measures to determine the risk of recurrent falls. Although our score performed well in our patient sample, an independent validation sample is important before incorporation into clinical practice.

Differences found in this study may be due to different methods applied by previous authors to identify risk factors associated with falls, different classification of fallers and nonfallers, and different sample characteristics. One limitation of this study is the retrospective classification of recurrent fallers and nonfallers. However, our rate of recurrent fallers was similar to that reported by previous prospective studies. We are currently carrying out a one-year prospective study and further analysis may differentiate predictors of single and recurrent falls.

## 5. Conclusions

The occurrence of falls in PD is high and recurrent falls, especially, can play an important role in the functional status of patients. The identification of risk factors associated with recurrent falls in people with PD, especially those that are modifiable, could be useful to target intervention programs to improve balance and possibly reduce the risk of falls and related injuries. We encourage the use of the UPDRS-ADL section, BBS, and FES-I in screening for falls risk in individuals with PD, measures that could be easily performed either in a home or outpatient clinical setting.

## Figures and Tables

**Table 1:** Demographic, disease-specific characteristics and balance-related measures of the sample and comparison between recurrent fallers and nonfallers.

Characteristics	Recurrent fallers (*n* = 52)	Nonfallers (*n* = 119)	*P* value
	Median (min–max)	Median (min–max)	
Age (years)	71 (61; 85)	70 (55; 94)	0.355^*^
Disease duration (years)	8.5 (1; 31)	4 (1; 20)	<0.001^*^
LED (mg)	775 (300; 1500)	500 (300; 1621.9)	<0.001^*^
Modified Hoehn and Yahr	3 (2; 4)	2.5 (1; 4)	<0.001^*^
UPDRS ADL	19 (2; 29)	9 (0; 24)	<0.001^*^
UPDRS Motor	43 (13; 65)	25.5 (7; 59)	<0.001^*^
Schwab and England (%)	70 (40; 100)	90 (40; 100)	<0.001^*^
PDQ-8	42.2 (0; 93.7)	25 (0; 78.1)	<0.001^*^
FES-I	40 (23; 62)	27 (16; 59)	<0.001^*^
ABC (%)	37.2 (1.9; 81.9)	63.1 (16.2; 100)	<0.001^*^
BBS	42 (27; 54)	52 (37; 56)	<0.001^*^
DGI	16 (8; 22)	21 (10; 24)	<0.001^*^
FRT (centimeters)	14.5 (7; 32)	20.5 (6; 34)	<0.001^*^
TUG (seconds)	20.2 (7.9; 149.5)	14.2 (7.0; 36.4)	<0.001^*^

^*^Mann-Whitney test.

LED: levodopa equivalent dose; UPDRS: Unified Parkinson's Disease Rating Scale; ADL: activities of daily living; PDQ-8: eight-item Parkinson's Disease Questionnaire; FES-I: Falls Efficacy Scale-International; ABC: Activities-specific Balance Confidence Scale; BBS: Berg Balance Scale; DGI: Dynamic Gait Index; TUG: Timed Up and Go Test; FRT: Functional Reach Test.

**Table 2:** Multivariate logistic regression analysis of the sample (*n* = 164).

Variables	Odds ratio (95% CI)	*P* value
LED (per 100 mg increase)	1.283 (1.092–1.507)	0.002
BBS ≤ 48 points	3.868 (1.175–12.738)	0.026
FES-I > 30 points	5.956 (1.57–22.598)	0.009
UPDRS-ADL > 16 points	10.042 (3.568–28.264)	<0.001

CI: confidence interval; LED: levodopa equivalent dose; UPDRS-ADL: *Unified Parkinson's Disease Rating Scale-*activities of daily living; FES-I: Falls Efficacy Scale-International; BBS: Berg Balance Scale.
